# Resolution of the High versus Low debate for Old and Middle Kingdom Egypt

**DOI:** 10.1371/journal.pone.0314612

**Published:** 2025-05-28

**Authors:** Pınar Erdil, Lyndelle Webster, Margot Kuitems, Christian Knoblauch, Laurel Bestock, Felix Höflmayer, Hans Beeckman, Dorian Q. Fuller, Sturt W. Manning, Michael W. Dee

**Affiliations:** 1 Centre for Isotope Research, Energy and Sustainability Research Institute Groningen, University of Groningen, Groningen, The Netherlands; 2 Groningen Institute of Archaeology, University of Groningen, Groningen, The Netherlands; 3 Austrian Academy of Sciences, Austrian Archaeological Institute, Vienna, Austria; 4 Department of Heritage, History and Classics, Swansea University, Wales, United Kingdom; 5 Joukowsky Institute for Archaeology and the Ancient World, Brown University, Providence, United States of America; 6 Service of Wood Biology, Royal Museum for Central Africa, Tervuren, Belgium; 7 Institute of Archaeology, University College London, London, United Kingdom; 8 Cornell Tree Ring Laboratory, Department of Classics, Cornell University, Ithaca, New York, United States of America; 9 The Cyprus Institute, Aglantzia, Cyprus; University of Bern, Institute of Forensic Medicine, SWITZERLAND

## Abstract

The unrivaled millennia-long historical chronology of ancient Egypt forms the backbone for archaeological synchronization across the entire Eastern Mediterranean region c. 3000-1000 BCE. However, for more than a century, scholars have wrangled over the correct calendrical positioning of this record, with older scenarios being referred to as ‘High’, and younger ones, ‘Low’ chronologies. Offsets between the two can be as great as a century, substantially confusing connections with other civilizations of the time. Here, we settle this debate for two major periods of political unity in ancient Egypt, the Old Kingdom (the Pyramid Age), and the Middle Kingdom. We introduce 48 high-precision radiocarbon dates obtained through rare access to museum collections as well as freshly excavated samples. By combining these new results with legacy radiocarbon data and with text records for reign lengths of kings within a Bayesian statistical framework, we show that the Low Chronology is no longer empirically supported for the Old and Middle Kingdoms, and resolve a long-standing historical schism.

## Introduction

Being one of the most enduring of the early civilizations, establishing a reliable chronology for Egypt has been a major goal for historians. A unified chronology would not only be advantageous for dating events in Egypt itself, but also for resolving cause and effect relationships in political and cultural interactions across the wider region [1–3]. Specifically, the Old Kingdom (OK, c. mid-late 3^rd^ millennium BCE, the ‘Pyramid Age’) and Middle Kingdom (MK, c. late 3^rd^/early 2^nd^ millennium BCE) periods provide the key pillar for placing and linking the chronology of the Early and Middle Bronze Ages in the ancient Near East and Eastern Mediterranean [4–8]. Many attempts have been made to create a robust and coherent timeline for this region [9–13]. Often the Minoan eruption of Thera (Santorini) has been used as a universal time marker, due to its impact on Greece, Anatolia and the wider Eastern Mediterranean; however, the absolute date of the eruption itself remains highly controversial [14–20].

Currently, most Egyptian history is underpinned by astronomical data, king-lists, and other official records on papyri and stone [21], which are open to multiple interpretations and have led to chronological ambiguity. The astronomical data include records of the rising of stars, lunar events, and various other celestial phenomena [22–27]. Amongst these, the most important are the so-called Sothic records, which describe the heliacal rising of the star Sirius (Egyptian Sopdet; Greek Sothis). This event marked the first day of the ancient Egyptian civil calendar [1,28]. Sothic dates are, on rare occasions, recorded alongside the regnal year of the contemporary Egyptian king, making these documents invaluable to chronological research. One of the earliest Sothic records comes from the Illahun Papyrus assigned to Year 7 of king Senusret III of the MK, a ruler known for military expeditions to Nubia and the Levant [1,25,27–32]. Different calendrical dates have been proposed for this document. For instance, Parker [22] calculated that this observation took place in 1872 BCE, thereby anchoring Senusret III’s reign and the long contiguous MK sequence precisely in time. This positioning has become known as the High Chronology. The same record was later dated by Krauss [33] to 1830 BCE using a different interpretation of where the observation was made. In combination with generally shorter reign lengths, this younger (lower) position of the Sothic date is pivotal to the Low Chronology. The correctness of these two alternatives, High and Low, has been actively debated for more than a century [1,26,29,30,34,35].

A similar dilemma exists for the OK, but here the chronology remains truly floating in time. The main reason is that no reliable Sothic records are available from the OK, although other astronomical linkages have been attempted [27,36]. Some scholars have argued for the possibility of Sothic-dated stone vessels from Dynasty V (OK); although these interpretations also remain speculative and open to debate [26,37].

Radiocarbon (^14^C) dating has been applied to ancient Egyptian history ever since the development of the method [38] and it continues to contribute to understanding of the civilization. Examples include the results obtained on the coffin of the famous king Tutankhamun and the funerary boat of king Khufu adjacent to the Great Pyramid of Giza [39–41]. However, some of these early studies produced results which diverged from historical expectations by hundreds of years. These offsets were caused by a variety of reasons, such as differences between the inherent and historical age of specific items (so-called inbuilt age), insufficient removal of contaminants, or ambiguity in the archaeological attributions [42]. The resolution of many of these discrepancies was important to the establishment of ^14^C dating as the pre-eminent tool in chronological research.

The most significant application thus far of ^14^C dating and Bayesian modeling to ancient Egypt was published by Bronk Ramsey et al. [43]. Their study utilized 211 ^14^C dates from a wide range of Egyptian contexts linked with specific kings, and, combined with text data on the reign-lengths of these rulers, resulted in a science-based absolute chronology. While this analysis accurately positioned the New Kingdom (NK, c. mid-late 2^nd^ millennium), a conclusive result for the OK and MK was not achieved, mainly due to the limited amount of relevant sample material available for these periods. For example, an almost equivalent probability was still allocated to both High and Low Chronologies for many royal accession dates of the MK. The situation for the OK was even more unsatisfactory, with the position of this 500-year contiguous sequence defined only by 17 ^14^C dates. Consequently, several key chronological debates relating to the OK and MK remained unresolved.

Since this time, there have been significant developments in the field of ^14^C dating. First, a new iteration of the Northern Hemisphere calibration curve, the mathematical function used to convert raw ^14^C data into calendar time ranges, has recently been released that offers higher accuracy and precision than ever before (IntCal20) [44]. This update includes additional reference data over the mid-2^nd^ millennium BCE, which especially enables the MK period to be studied at higher resolution. A further refinement concerns a regional offset to the calibration curve proposed for ancient Egyptian materials [45]. This small-scale discrepancy, often known as the ‘seasonal effect’, was attributed to a local growing season or latitudinal effect, perhaps modulated by changing climatic regimes [46]. Its magnitude was initially estimated to be 19 ± 5 ^14^C yr BP, a figure obtained by comparing a dataset of known-age annual samples that were in the inundation zone of the Nile with the IntCal04 curve [45]. However, recent analyses against IntCal20 have revised it down to 12 ± 5 ^14^C yr BP [47].

In our study, 160 ^14^C dates (48 new and 112 previously published) are employed in a new suite of Bayesian chronological models for the OK and MK in order to produce the most substantive science-based chronology ever compiled for these two periods. Our results indicate that, for the first time, we can define a single coherent position for this pivotal historical sequence.

## Materials and methods

New samples for ^14^C dating were collected from secure ancient Egyptian contexts relating to OK and early (Dynasty XII) MK periods. For the OK, permissions were gained to sample short-lived organic materials with known archaeological provenance from the collections of the Natural History Museum, London; the Petrie Museum of Egyptian Archaeology, London; World Museum, Liverpool; and Cambridge University (listed in Table 1). The samples were pretreated using the routine procedures of [52] and dated at the Oxford Radiocarbon Accelerator Unit (ORAU) (see S1 File).

**Table 1:** The new short-lived plant samples and ^14^C dates from the Old Kingdom period of Egyptian history.

Collection		Material	Site	Historical Assignment	Basis for Historical Assignment	Laboratory Code	^14^C Date		Calibrated Date Range (BCE, 95%)		∂^13^C (‰, VPDB)
Name	Accession No.						^14^C yr BP	± 1σ	From	To	
World Museum, Liverpool	50.33	Bone	Nuwayrat	Between Sekhemkhet and Khufu	Museum records. Also B. Vanthuyne (site excavator)	OxA-33186	4093	35	2866	2496	-18.9
World Museum, Liverpool	50.33	Textile	Nuwayrat	Excluded (Modern)	Museum records. Also B. Vanthuyne (site excavator)	*OxA-33187*	*10*	*27*	*1696 CE*	*1915 CE*	*-26.8*
Petrie Museum, London	UC31180	Linen	Deshasheh	Dynasty V or VI	Museum records. Also Petrie [48]	OxA-32270	3815	38	2452	2141	-24.6
Petrie Museum, London	UC31181	Linen	Deshasheh	Dynasty V or VI	Museum records. Also Petrie [48]	OxA-32271	3899	37	2473	2210	-24.7
Cambridge University	UN.C	Plant remains	Pyramid of Unas, Saqqara	Reign of Unas	Departmental records	OxA-X-2555-51	3980	120	2875	2151	-22.8
Petrie Museum, London	UC31182	Linen	Deshasheh	Dynasty V or VI	Museum records Also Petrie [48]	OxA-30209	3915	29	2471	2296	-25.4
Petrie Museum, London	UC32772	Papyrus	Pyramid of Neferirkare, Abusir	Reign of Neferirkare	Museum records. Also Posener-Kriéger & de Cenival [49]	OxA-30539	4010	60	2852	2343	-8.9
Petrie Museum, London	UC55050	Linen	Pyramid of Pepy I Saqqara	Reign of Pepy I	Museum records	OxA-30211	3956	32	2571	2344	-24.6
Petrie Museum, London	UC55051	Linen	Pyramid of Merenre, Saqqara	Reign of Merenre	Museum records	OxA-30028	3968	31	2574	2349	-24.3
Natural History Museum, London	HR 10048 (16.0925)	Bone	Cemetery F, Grave 243, Abydos	Dynasty VI	Museum records Also Leonard and Loat [50] Yamamoto [51]	OxA-30874	3918	33	2557	2291	-18.8
Natural History Museum, London	HR 10049 (16.0926)	Bone	Cemetery F, Grave 69, Abydos	Dynasty VI	Museum records Also Leonard and Loat [50] Yamamoto [51]	OxA-30875	3871	33	2463	2209	-18.9
Natural History Museum, London	HR 10054 (16.0946)	Bone	Cemetery F, Grave 34, Abydos	Dynasty VI	Museum records Yamamoto [51]	OxA-30876	3910	32	2473	2291	-18.2

For MK, short-lived plants and tree rings from two timber beams (acacia, *Vachellia tortilis*, FK-001-01 and FJ-001-04) were sampled from the fortress of Uronarti, Sudan. The samples were obtained by the Uronarti Regional Archaeological Project (URAP) with the official permission and collaboration of the National Corporation of Antiquities and Museums, Sudan. The new excavations have confirmed that the construction of the fortress took place during the reign of Senusret III [53]. The short-lived organic materials on the site have a clear stratigraphic sequence which includes the materials from the earliest wall structures and foundation layers for the construction of the fortress (Unit FA) as well as subsequent occupation layers (Unit FI) [53] (see Table 2).

**Table 2:** The new short-lived plant samples and ^14^C dates from Uronarti (Sudan) in the MK period of Egyptian history, listed stratigraphically from oldest to youngest.

ID	Material	Stratigraphy at the Site	Laboratory Code	^14^C Date		Calibrated Date Range (BCE, 95%)	
				^14^C yr BP	± 1σ	From	To
FA-027-6/7	palm	Founding phase	GrM-30065	3527	24	1939	1753
FA-027-6	*Hordeum vulgare*	Founding phase	GrM-20337	3559	26	2015	1776
FA-001-2	rhizome, cereal	Founding phase	GrM-20338	3559	26	2015	1776
FJ-001-7	reed/grass culm- cf. *Phragmites* type	Founding phase	GrM-30033	3642	40	2136	1899
FK-002-2	reed/halfa grass culm- *cf. Demostachya bipnnata* type	Founding phase	GrM-30069	3553	24	2008	1775
FA-042-10	*Hordeum vulgare*	FA Phase 2a	GrM-20336	3551	26	1939	1767
FA-042-10B	reed/halfa grass culm- *cf. Demostachya bipinnata* type	FA Phase 2a	GrM-30066	3528	23	2009	1773
FA-033-8	desiccated barley: *Hordeum hexastichum*	FA Phase 2b	GrM-30031	3640	40	2136	1897
FA-032-7	*Hordeum vulgare*	FA Phase 2b	GrM-20335	3566	26	2018	1778
FA-031-4	desiccated barley: *Hordeum hexastichum*	FA Phase 2b	GrM-30032	3695	40	2201	1959
FA-035-5	desiccated barley: *Hordeum hexastichum*	FA Phase 3b	GrM-30068	3610	27	2108	1888
FA-035-1	*Hordeum vulgare*	FA Phase 3b	GrM-21296	3570	75	2137	1697
FI-001-92A	reed/grass culm- *cf. Phragmites* type	FI Lot 7 upper	GrM-30071	3542	24	1952	1771
			GrM-30094	3557	26	2014	1775
FI-001-91B	reed/halfa grass culm- *cf. Demostachya bipinnata* type	FI Lot 6	GrM-30072	3543	24	1954	1772
FI-001-91A	desiccated christ’s thorn: *Ziziphus cf. spina-christi*	FI Lot 6	GrM-30093	3535	24	1946	1769
FI-001-90	desiccated christ’s thorn: *Ziziphus cf. spina-christi*	FI Lot 5 upper	GrM-30070	3541	24	1951	1771
FI-001-88	desiccated christ’s thorn: *Ziziphus cf. spina-christi*	FI Lot 4 upper	GrM-30073	3462	24	1881	1692
FI-001-86B	desiccated christ’s thorn: *Ziziphus cf. spina-christi*	FI Lot 2 upper	GrM-30067	3494	24	1889	1744

Additional care was taken to sample the timber beams from the base (primary) levels of the fortress (S1 Fig). As far as the archaeological evidence suggests, there was no reconstruction of the main fortification walls of the fortress, which were the first features to be built [53]. It is thus reasonable to assume that the wood beams date to the original construction of the fortress. The details of the Uronarti specimens are listed in Tables 2 and 3 and further discussed in S1 File.

**Table 3:** The tree-ring ^14^C sequences from the fortress of Uronarti (Sudan) in the MK period of Egyptian history.

ID	Tree-ring Sequence	Laboratory Code	^14^C Date		Calibrated Date Range (BCE, 95%)		∂^13^C (‰, VPDB)
			^**14**^ **C yr BP**	**± 1σ**	**From**	**To**	
FK-001-01	Bark Edge (0)	GrM-22702	3509	19	1893	1750	-27.3
	(-1)	GrM-22703	3540	19	1944	1774	-26.6
	(-2)	GrM-22705	3532	19	1935	1772	-25.8
	(-3)	GrM-22706	3532	19	1935	1772	-25.6
	(-4)	GrM-22707	3533	19	1936	1772	-26.8
	(-5)	GrM-22708	3568	19	2014	1826	-25.8
		GrM-22709	3570	19	2014	1827	-25.6
	(-6)	GrM-22710	3553	19	1956	1776	-25.8
	(-7)	GrM-22713	3544	19	1947	1775	-25.5
	(-8)	GrM-22714	3547	19	1950	1775	-25.4
	(-9)	GrM-22715	3538	19	1942	1773	-25.8
FJ-001-04	Bark Edge (0)	GrM-24029	3558	20	2008	1778	-25.6
	(-1)	GrM-24035	3546	19	1949	1775	-26.4
	(-2)	GrM-24034	3589	31	2032	1826	-25.9
	(-3)	GrM-24030	3529	19	1933	1771	-25.1
	*(-4)*	*(failed)*	–	–	–	–	–
	(-5)	GrM-24031	3560	19	2009	1779	-24.1
		GrM-24042	3600	38	2127	1783	-23.8
	(-6)	GrM-24032	3564	23	2015	1779	-23.6

Tree rings were subjected to the α-cellulose extraction protocol, whereas the short-lived samples were pretreated using routine holocellulose extraction protocol [54]. These samples were graphitized and measured by accelerator mass spectrometry (AMS) at the Centre for Isotope Research (CIO), Groningen. The resulting ^14^C ages are incorporated into revised Bayesian models for the OK and MK using the OxCal software [55,56] version 4.4 with IntCal20 [44].

All necessary permits were obtained for all the new samples mentioned above, which complied with all relevant regulations. Additional information regarding the ethical, cultural, and scientific considerations specific to inclusivity in global research is included in the Supporting Information (S2 File, Checklist).

## Results

The new ^14^C results and their calibrated date ranges are listed in Tables 1-3. One measurement from the OK dataset turned out to be modern and was excluded from the remainder of the analysis, and one sample from the MK data set failed due to a low carbon yield. Three samples were pretreated and measured in duplicate and showed excellent agreement (GrM-22708 and GrM-22709, χ^2^ = 0.01 versus 3.8, df = 1; GrM-24031 and GrM-24042, χ^2^ = 0.89 versus 3.8, df = 1; GrM-30071 and GrM-30094, χ^2^ = 0.18 versus 3.8, df = 1) [57].

A considerable proportion of our data set (including 37 new ^14^C dates) is associated with the pivotal reign of Senusret III. Many of these were individual tree rings from structural beams, recently excavated from this king’s fortress at Uronarti in Nubia (modern-day Sudan, see S1 File).

A collection of 18 Bayesian statistical models were produced for the OK and MK, taking into account several potential parameters such as multiple reign-length scenarios [4,21,25,26, 58, 59] and varying applications of a seasonal effect. The rationale for, and the link to, the codes for all these models can be found in the S1 File. A summary of key modeled dates is given in Table 4.

**Table 4:** The date ranges from the OK and MK models P1, P2 and P3. These models incorporate different reign length configurations by refs. Hornung et al. [21] (model P1), Kitchen [4] (model P2), Shaw [59] (model P3).Outputs of accession dates for individual OK and MK kings are given in the S2 and S3 Tables. All dates in BCE.

	Modelled Dates											
	OK & MK P1				OK & MK P2				OK & MK P3			
	(68% hpd)		(95% hpd)		(68% hpd)		(95% hpd)		(68% hpd)		(95% hpd)	
	From	To	From	To	From	To	From	To	From	To	From	To
Start of OK	2679	2642	2698	2629	2695	2652	2750	2635	2686	2648	2704	2632
Start of FIP	2267	2228	2283	2204	2220	2183	2236	2158	2247	2217	2262	2197
Start of MK	2058	2045	2065	2037	2058	2045	2064	2038	2063	2048	2070	2040
End of MK	1819	1806	1825	1799	1819	1807	1825	1800	1811	1794	1818	1784

## Discussion

**Fig 1:**
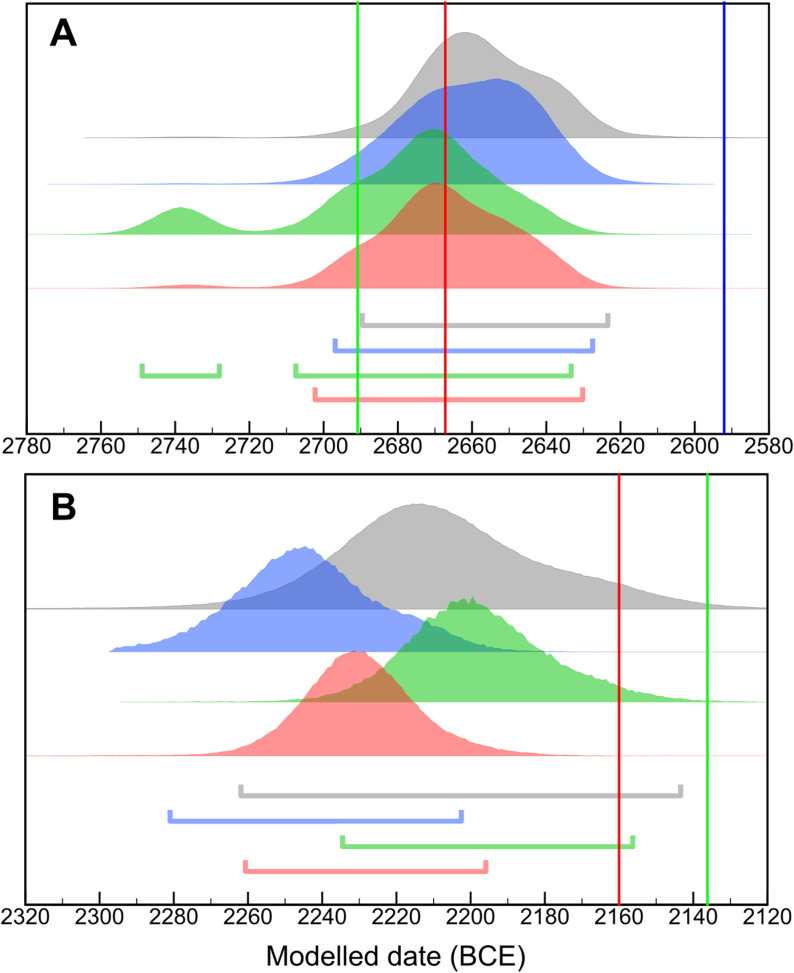
The results from the OK models compared to Bronk Ramsey et al. [43] **(grey)** **(A) the start of the OK, (B) the start of the First Intermediate Period (FIP).** The probability density functions shown represent 3 different OK models which incorporate different reign length interpretations: Hornung et al. [21] (blue), Kitchen [4] (green), Shaw [59] (red) with horizontal bars indicating the 95.4% range. Using the same color codes of reign length assumptions, the vertical lines demarcate the absolute dates for the start of the OK and FIP from each of these historical chronologies, where available (the Hornung et al. [21] estimate lies off the scale ~ 2118 BCE).

The results we obtained for the OK were consistent with earlier ^14^C studies. Even though our chronological models are the most comprehensive ever constructed for this period (33 ^14^C dates), our results act to reaffirm the High chronological estimates achieved previously (Fig 1) [43]. Indeed, the Low chronological position (blue vertical line in Fig 1) [21] is ruled out at 95% probability. A key strength of our data set is that it was distributed evenly across the OK, whereas previous models for the period were largely cantilevered out from Dynasty III and IV on the basis of reign-length estimates. Our results also continue to underscore the congruence between the 4.2 ka aridification event (c. 2250 BCE) and the political fragmentation that concluded the OK (instigating the First Intermediate Period, FIP) [60,61]. Some scholars contend that the centralized state was already irreparably damaged by the time this climatic downturn set in [62,63], but such adverse conditions would have compounded the challenges at hand.

**Fig 2:**
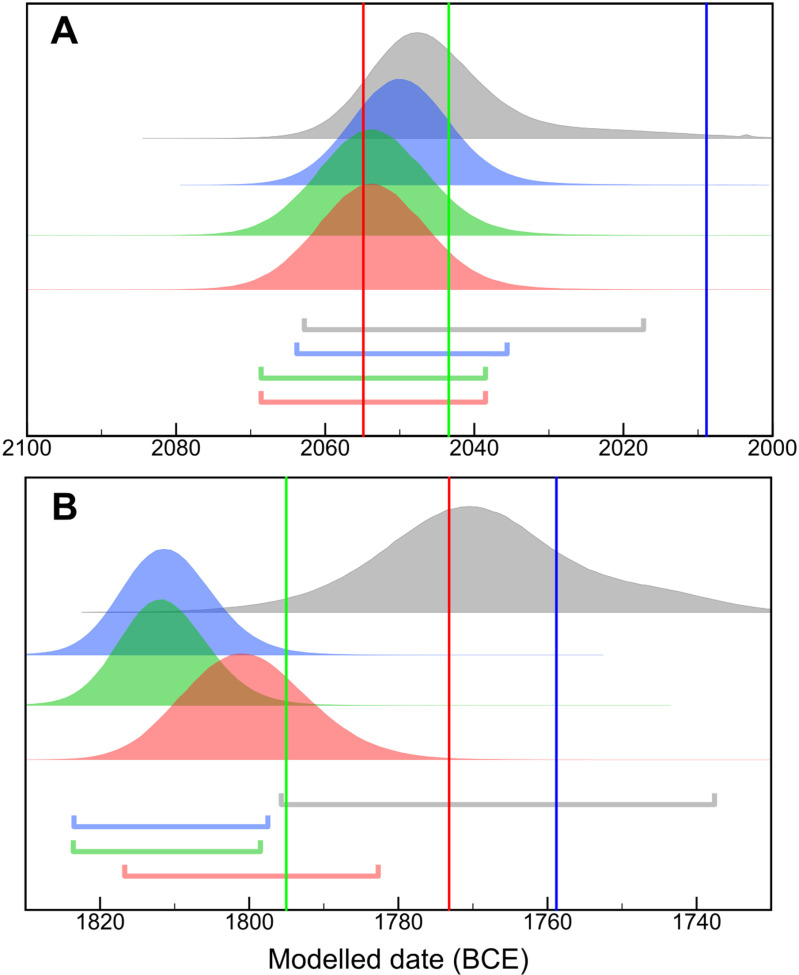
The results from the MK models compared to Bronk Ramsey et al. [43] **(grey)** **(A) the start of the MK (B) the end of the MK.** The probability density functions shown represent 3 different MK models which incorporate different reign length interpretations: Hornung et al. [21] (blue), Kitchen [4] (green), Shaw [59] (red) with horizontal bars indicating the 95.4% range. Using the same color codes of reign length assumptions, the vertical lines demarcate the absolute dates for the start and end of MK from each of these historical chronologies, where available.

**Fig 3:**
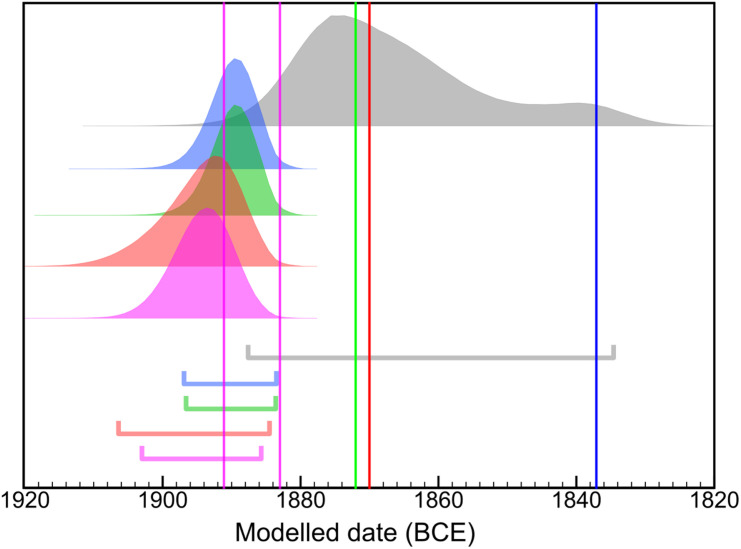
The results for the accession of king Senusret III compared to Bronk Ramsey et al. **[43] (grey) calibrated using IntCal20.** The probability density functions shown represent 4 different MK models which incorporate different reign length interpretations: Hornung et al. [21] (blue), Kitchen [4] (green), Shaw [59] (red) and Gautschy [25] (two options, magenta) with horizontal bars indicating the 95.4% range. Using the same color codes of reign length assumptions, the vertical lines demarcate the absolute dates for the start of the reign of Senusret III from each of these historical chronologies, where available.

Unlike all previous studies, our MK models (comprising 127 ^14^C dates) are able to distinguish between the High and Low chronological scenarios. Irrespective of the reign-length configuration employed, the models exclusively support the High chronological positions. Our estimates for the beginning and end of the MK are of unprecedented precision, with the latter juncture shifted several decades earlier than many previous estimations (Fig 2). Specifically, we highlight our results for the accession date of king Senusret III on Fig 3. It was during his reign that the decisive Sothic observation was documented. Confirming the early 19^th^ century BCE age of this tie-point is not only crucial to the MK but to the dating of other events of the ancient Near East and Eastern Mediterranean. In fact, our results for the accession dates of most MK rulers peak 10-15 years earlier than even most High Chronology estimates. This pattern is evident with or without the application of the seasonal offset (S2 Fig). In short, our results align the entire MK sequence with the High Chronology and provide no empirical support for the Low chronological positioning. The resolution of this important timeline raises the prospect of a more coherent and integrated approach to studying this defining region of the ancient past.

## Conclusions

As an essential tool in archaeological research, ^14^C dating has continuously contributed to our understanding of ancient Egyptian chronology and recent advancements in the field offer even greater insight. In particular, the increase in research on single tree rings has resulted in improved calibration curves and enabled ever finer resolution, which in turn are allowing us to resolve long-standing chronological debates.

In this paper, we present an updated chronology for the OK and MK. Our conclusions rely on the most recent data and assumptions, including the latest calibration records, reign orders and lengths. Because of the Bayesian approach taken, any major revisions to these sources of prior information would lead to alternative conclusions. Nonetheless, the results that we present here offer the most refined synthesis possible on the basis of existing data and methods.

In sum, our findings reaffirm earlier ^14^C-based studies for the OK, and rule out the Low Chronology for the MK. This offers more reliable synchronization with other contemporary ancient Near Eastern and Eastern Mediterranean civilizations.

## Supporting information

S1 Fig Wooden support beams were found within the remains of the Uronarti fortress. (A) A typical example of the adequate state of preservation of a wooden beam. (B) The size of the beams compared with the mudbrick work. (C) The red arrow indicates the location of a beam within the fortress wall. (Photos by Lyndelle Webster).
(PNG)

S2 Fig The results for the accession date of king Senusret III from various MK models compared to Bronk Ramsey et al. [43] (grey) using IntCal20 without any seasonal effect applied. The resulting probability density functions of 4 different MK models which incorporate different reign length interpretations: Hornung et al. [21] (blue), Kitchen [4] (green), Shaw [59] (red), Gautschy [25] (two options, magenta) with horizontal bars indicating the 95.4% range. Vertical lines indicate the traditional absolute dates for the accession of Senusret III, using the same colours for the respective reign length schemes.
(PNG)

S3 Fig The results for Dynasty XII rulers from the MK P4 model. 95.4% probability ranges are shown. Traditional absolute dates for the rulers’ accession based on High [59] and Low [21] chronologies is shown for comparison as vertical bars (red and blue respectively). The probability density functions when the model is calibrated against IntCal20.
(PNG)

S1 Table The Illahun papyri dated by Bronk Ramsey et al. [43], and two published results on the Illahun Sothic Papyrus (OxA-23170 and OxA-23171) [32] listed stratigraphically. Our models incorporated the prior assumption that these documents could be regarded as forming a relative sequence in each reign, based on the inscribed regnal years.
(PDF)

S2 Table The modelled 95% ranges for the OK rulers. Models calibrated with IntCal20.
(PDF)

S3 Table The modelled 95% ranges for the MK rulers. Models calibrated with IntCal20.
(PDF)

S1 File Supplementary text on materials and methods. (DOCX)

S2 File Inclusivity in global research checklist. (DOCX)

## References

[1] Ward WA. The present status of Egyptian chronology. Bull Am Schools Orient Res. 1992;288:53–66.

[2] Shortland AJ. An introduction to Egyptian historical chronology. In: Shortland AJ, Bronk Ramsey C, editors. Radiocarbon and the chronologies of ancient Egypt. Oxford: Oxbow Books; 2013. pp. 37–50.

[3] Höflmayer F. Radiocarbon dating and Egyptian chronology—From the “Curve of Knowns” to Bayesian modeling. In: The Oxford handbook of topics in archaeology. Oxford: Oxford Academic; 2016.

[4] Kitchen KA. Regnal and genealogical data of ancient Egypt (absolute chronology I) the historical chronology of ancient Egypt, a current assessment. In: Bietak M, editor. The synchronisation of civilizations in the Eastern Mediterranean in the second millennium B.C. Proceedings of an International Symposium at Schloss Haindorf, 15th-17th November 1996 and at the Austrian Academy, Vienna, 11th-12th May 1998. Vienna: Austrian Academy of Sciences Press; 2000. pp. 39–52.

[5] Bietak M, Höflmayer F. Introduction: High and low chronology. In: Bietak M, Czerny E, editors. The synchronisation of civilizations in the Eastern Mediterranean in the second millennium B.C. III. Vienna: Austrian Academy of Sciences Press; 2007. pp. 13–23.

[6] Höflmayer F, Cohen SL. Chronological conundrums: Egypt and the Middle Bronze Age Southern Levant. JAEI. 2017;13:1–6.

[7] Höflmayer F, Manning SW. A synchronized early Middle Bronze Age chronology for Egypt, the Levant, and Mesopotamia. J Near East Stud. 2022;81:1-24.

[8] Höflmayer F. Establishing an absolute chronology of the Middle Bronze Age. In: Radner K, Moeller N, Potts DT, editors. The Oxford History of the Ancient Near East, Volume II: From the end of the third millennium BC to the fall of Babylon. Oxford: Oxford University Press; 2022.

[9] Bietak M, editor. The synchronisation of civilizations in the Eastern Mediterranean in the second millennium B.C. Proceedings of an International Symposium at Schloss Haindorf, 15th-17th November 1996 and at the Austrian Academy, Vienna, 11th-12th May 1998. Vienna: Austrian Academy of Sciences Press; 2000.

[10] Bietak M, editor. The synchronisation of civilizations in the Eastern Mediterranean in the second millennium B.C. II. Vienna: Austrian Academy of Sciences Press; 2003.

[11] Manning SW, Bronk Ramsey C, Kutschera W, Higham T, Kromer B, Steier P, et al. Chronology for the Aegean Late Bronze Age 1700-1400 B.C. Science. 2006;312:565–9. 16645092 10.1126/science.1125682

[12] Manning SW, Griggs CB, Lorentzen B, Barjamovic G, Ramsey CB, Kromer B, et al. Integrated tree-ring-radiocarbon high-resolution timeframe to resolve earlier second millennium BCE Mesopotamian chronology. PLoS One. 2016;11(7):e0157144. doi: 10.1371/journal.pone.0157144 27409585 PMC4943651

[13] Bietak M, Czerny E, editors. The synchronisation of civilizations in the Eastern Mediterranean in the second millennium B.C. II: Proceedings of the SCIEM 2000–2nd EuroConference, Vienna, 28th of May – 1st of June 2003.Vienna: Austrian Academy of Sciences Press; 2007.

[14] Friedrich WL, Kromer B, Friedrich M, Heinemeier J, Pfeiffer T, Talamo S. Santorini eruption radiocarbon dated to 1627-1600 B.C. Science. 2006;312:548. 16645088 10.1126/science.1125087

[15] Manning SW, Höflmayer F, Moeller N, Dee MW, Bronk Ramsey C, Fleitmann D, et al. Dating the Thera (Santorini) eruption: archaeological and scientific evidence supporting a high chronology. Antiquity. 2014;88:1164–79.

[16] Kutschera W. On the enigma of dating the Minoan eruption of Santorini. Proc Natl Acad Sci U S A. 2020;117(16):8677–9. doi: 10.1073/pnas.2004243117 32291333 PMC7183194

[17] Manning SW. Second Intermediate period date for the Thera (Santorini) eruption and historical implications. PLoS One. 2022;17(9): e0274835. doi: 10.1371/journal.pone.0274835 36126026 PMC9488803

[18] Pearson C, Sigl M, Burke A, Davies S, Kurbatov A, Severi M, Cole-Dai J, Innes H, Albert PG, Helmick M. Geochemical ice-core constraints on the timing and climatic impact of Aniakchak II (1628 BCE) and Thera (Minoan) volcanic eruptions. PNAS Nexus. 2022;1. 10.1093/pnasnexus/pgac048 PMC9802406 36713327

[19] Pearson C, Sbonias K, Tzachili I, Heaton TJ. Olive shrub buried on Therasia supports a mid-16th century BCE date for the Thera eruption. Sci Rep. 2023;13(1):6994. doi: 10.1038/s41598-023-33696-w 37117199 PMC10147620

[20] Manning SW. Problems of dating spread on radiocarbon calibration curve plateaus: The 1620-1540 BC example and the dating of the Therasia olive shrub samples and Thera volcanic eruption. Radiocarbon. 2024;66:341–70.

[21] Hornung E, Krauss R, Warburton D, editors. Ancient Egyptian chronology. Brill; 2006.

[22] Parker RA. The calendars of ancient Egypt. University of Chicago Press; 1950.

[23] Parker RA. Ancient Egyptian astronomy. Philos Trans R Soc Lond A. 1974;276: 51–65.

[24] Krauss R, Warburton DA. The basis for the Egyptian dates. In: Warburton DA, editor. Time’s up! Dating the Minoan eruption of Santorini: acts of the Minoan Eruption Chronology Workshop, Sandbjerg November 2007. Aarhus University Press; 2007. pp. 125–44.

[25] Gautschy R. Lunar and Sothic data from the archive of el-Lahun revisited: chronology of the Middle Kingdom. In: Horn M, et al., editors. Current research in Egyptology 2010 proceedings of the eleventh annual symposium. Oxbow Books; 2011. pp. 53–61.

[26] Gautschy R. A new astronomically based chronological model for the Egyptian Old Kingdom. J Egypt Hist. 2017;10: 69–108.

[27] Spalinger A. Feasts and fights: Essays on Time in Ancient Egypt. Yale Egyptological Institute; 2018.

[28] de Jong T. The heliacal rising of Sirius. In: Hornung E, Krauss R, Warburton D, editors. Ancient Egyptian chronology. Brill; 2006. pp. 432-438.

[29] Borchardt L. Der zweite Papyrusfund von Kahun und die zeitliche Festlegung des mittleren Reiches der ägyptischen Geschichte. Z Ägyptische Sprache Altertumskunde. 1899;37: 89–103.

[30] Kitchen KA. The chronology of ancient Egypt. World Archaeology. 1991;23: 201–8.

[31] Schneider T. Das Ende der kurzen Chronologie: eine kritische Bilanz der Debatte zur absoluten Datierung des Mittleren Reiches und der Zweiten Zwischenzeit. Egypt and the Levant. 2008;18: 275–313.

[32] Marcus E, Dee MW, Bronk Ramsey C, Higham T, Shortland A. Radiocarbon verification of the earliest astro-chronological datum. Radiocarbon. 2016;58:735–9.

[33] Krauss R. Sothis- und Monddaten. Studien zur astronomischen und technischen Chronologie Altägyptens. Hildesheimer Ägyptologische Beiträge; 1985.

[34] Krauss R. Late Old Kingdom chronology–another model. Egypt and the Levant. 2021;31:293–300.

[35] Belmonte JA, José J. Astronomy and chronology. In: Belmonte JA, editor. Astronomy of ancient Egypt: a cultural perspective. Springer; 2023. pp. 467–529.

[36] Spence K. Ancient Egyptian chronology and the astronomical orientation of pyramids. Nature. 2000;408(6810):320–4. doi: 10.1038/35042510 11099032

[37] Habicht ME, Gautschy R, Siegmann R, Rutica D, Hannig R. A new Sothis rise on a small cylindrical jar from the Old Kingdom. Göttinger Miszellen. 2015;247: 41–50.

[38] Arnold JR, Libby WF. Age determinations by radiocarbon content; checks with samples of known age. Science. 1949;110(2869):678–80. doi: 10.1126/science.110.2869.678 15407879

[39] Stuckenrath R, Ralph EK. University of Pennsylvania Radiocarbon Dates VIII. Radiocarbon. 1965;7:187–99.

[40] Nakhla SM, Mohammed FM. Cairo natural radiocarbon measurements I. Radiocarbon. 1974;16:1–5.

[41] Derricourt RM. Radiocarbon chronology for Egypt and North Africa. J Near East Stud. 1971;30: 271–92.

[42] Dee MW, Rowland JM, Higham TF, Shortland AJ, Brock F, Harris SA. Synchronising radiocarbon dating and the Egyptian historical chronology by improved sample selection. Antiquity. 2012;86:868–83.

[43] Bronk Ramsey C, Dee MW, Rowland JM, Higham TF, Harris SA, Brock F, et al. Radiocarbon-based chronology for Dynastic Egypt. Science. 2010;328:1554–7. 20558717 10.1126/science.1189395

[44] Reimer PJ, Austin WE, Bard E, Bayliss A, Blackwell PG, Bronk Ramsey C, et al. The IntCal20 Northern Hemisphere radiocarbon age calibration curve (0–55 cal kBP). Radiocarbon. 2020;62:725–57.

[45] Dee MW, Brock F, Harris SA, Bronk Ramsey C, Shortland AJ, Higham TF, Rowland JM. Investigating the likelihood of a reservoir offset in the radiocarbon record for ancient Egypt. J Archaeol Sci. 2010;37:687–93.

[46] Manning SW, Dee MW, Wild EM, Bronk Ramsey C, Bandy K, Creasman PP, et al. High-precision dendro-14C dating of two cedar wood sequences from First Intermediate Period and Middle Kingdom Egypt and a small regional climate-related 14C divergence. J Archaeol Sci. 2014;46:401–16.

[47] Manning SW, Wacker L, Büntgen U, Bronk Ramsey C, Dee MW, Kromer B, et al. Radiocarbon offsets and old world chronology as relevant to Mesopotamia, Egypt, Anatolia and Thera (Santorini). Sci Rep. 2020;10(1):13785. doi: 10.1038/s41598-020-69287-2 32807792 PMC7431540

[48] Petrie WMF. Deshasheh 1897: fifteenth memoir of the Egyptian Exploration Fund. Egypt Exploration Fund; 1898.

[49] Posener-Kriéger P, de Cenival JL, editors. Hieratic Papyri in the British Museum. Trustees of the British Museum; 1968.

[50] Leonard W, Loat S. A sixth dynasty cemetery at Abydos. J Egypt Archaeol. 1923;9:161–3.

[51] Yamamoto K. A late Old Kingdom burial assemblage from Abydos: tomb F109, excavated by the EEF in 1908. In: Regulski I, editor. Abydos: The Sacred Land at the Western Horizon. Leuven; 2019.

[52] Brock F, Higham T, Ditchfield P, Bronk Ramsey C. Current pretreatment methods for AMS radiocarbon dating at the Oxford Radiocarbon Accelerator Unit (ORAU). Radiocarbon. 2010;52:103–12.

[53] Bestock L, Knoblauch C. Leather, mud and grain: the 2018 excavations in Uronarti fortress. Sudan & Nubia. 2020;24:31–42.

[54] Dee MW, Palstra SWL, Aerts-Bijma AT, Bleeker MO, De Bruijn S, Ghebru F. Radiocarbon dating at Groningen: new and updated chemical pretreatment procedures. Radiocarbon. 2020;62:63–74.

[55] Bronk Ramsey C. Radiocarbon calibration and analysis of stratigraphy: the OxCal program. Radiocarbon. 1995;37:425–30.

[56] Bronk Ramsey C. Bayesian analysis of radiocarbon dates. Radiocarbon. 2009;51: 337–60.

[57] Ward GK, Wilson SR. Procedures for comparing and combining radiocarbon age determinations: a critique. Archaeometry. 1978;20: 19–31.

[58] Murnane WJ. Ancient Egyptian coregencies. Studies in Oriental Civilization 40. The Oriental Institute; 1977.

[59] Shaw I. The Oxford history of ancient Egypt. Oxford University Press; 2000.

[60] Höflmayer F. The southern Levant, Egypt, and the 4.2 ka BP event. In: Meller H, Arz HW, Jung R, Risch R, editors. 2200 BC: a climatic breakdown as a cause for the collapse of the Old World? 7th Archaeological Conference of Central Germany, October 23-26, 2014 in Halle (Saale). Landesmuseum für Vorgeschichte; 2015. pp. 113–30.

[61] Younes MA, Bakry A. The 4.2 ka BP climate event in Egypt: integration of archaeological, geoarchaeological, and bioarchaeological evidence. Afr Archaeol Rev. 2022;39: 315–44.

[62] Butzer KW. Sociopolitical discontinuity in the Near East C. 2200 B.C.E.: scenarios from Palestine and Egypt. In: Dalfes HN, Kukla G, Weiss H, editors. Third millennium BC climate change and Old World collapse. Springer; 1997. pp. 245–96.

[63] Bárta M. Long term or short term? Climate change and the demise of the Old Kingdom. In: Kerner S, Dann R, Bangsgaard P, editors. Climate and ancient societies. Museum Tusculanum Press; 2015. pp. 177-96.

