# Supplementary figures and images for: Resolution of the High versus Low debate for Old and Middle Kingdom Egypt
Source: PLoS One. 2025 May 28;20(5):e0314612. doi: 10.1371/journal.pone.0314612 (PMC12119019; doi:10.1371/journal.pone.0314612)

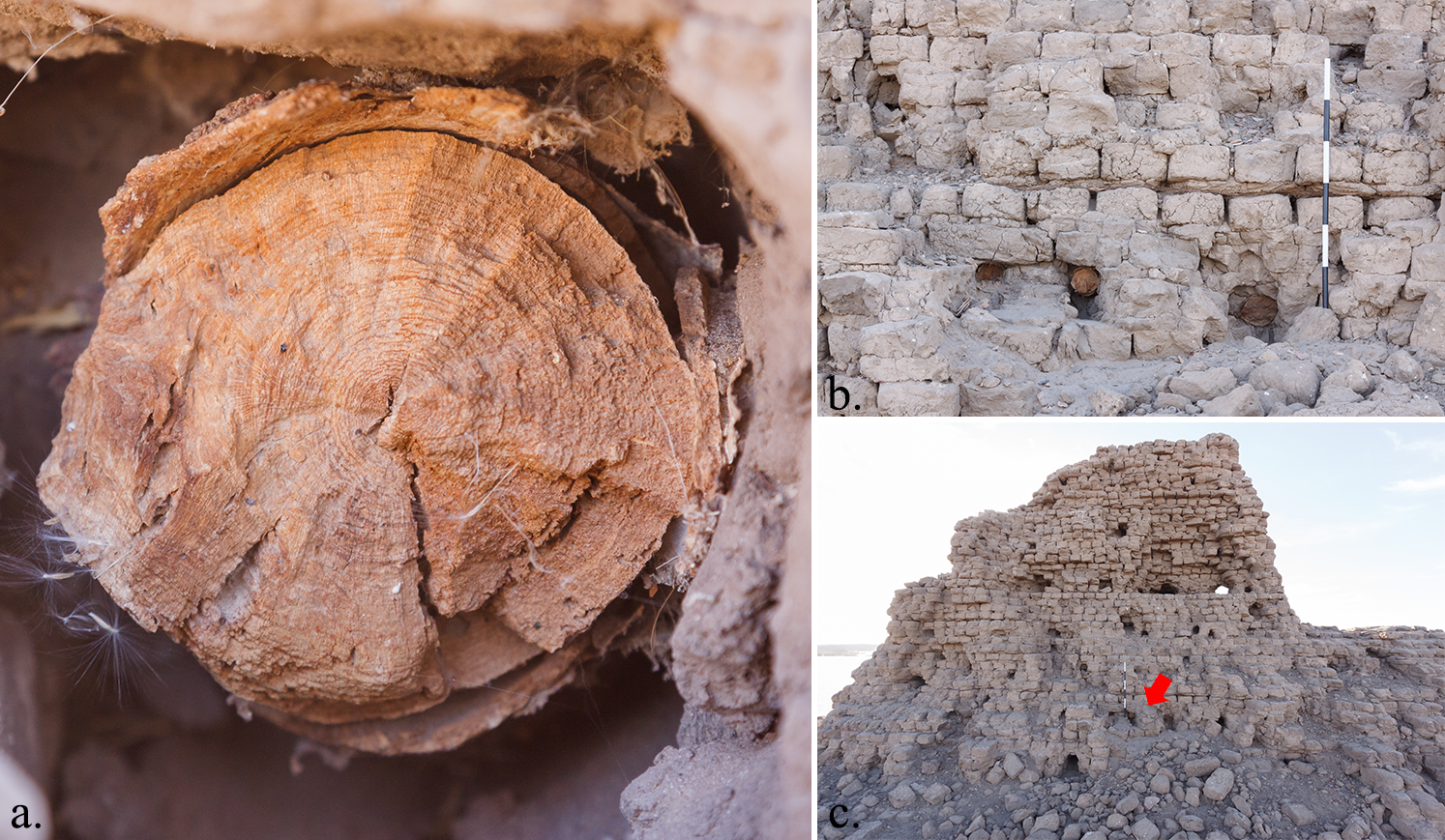

Supplement: S1 Fig — (A) A typical example of the adequate state of preservation of a wooden beam. (B) The size of the beams compared with the mudbrick work. (C) The red arrow indicates the location of a beam within the fortress wall. (Photos by Lyndelle Webster). (PNG) [file pone.0314612.s001.png]

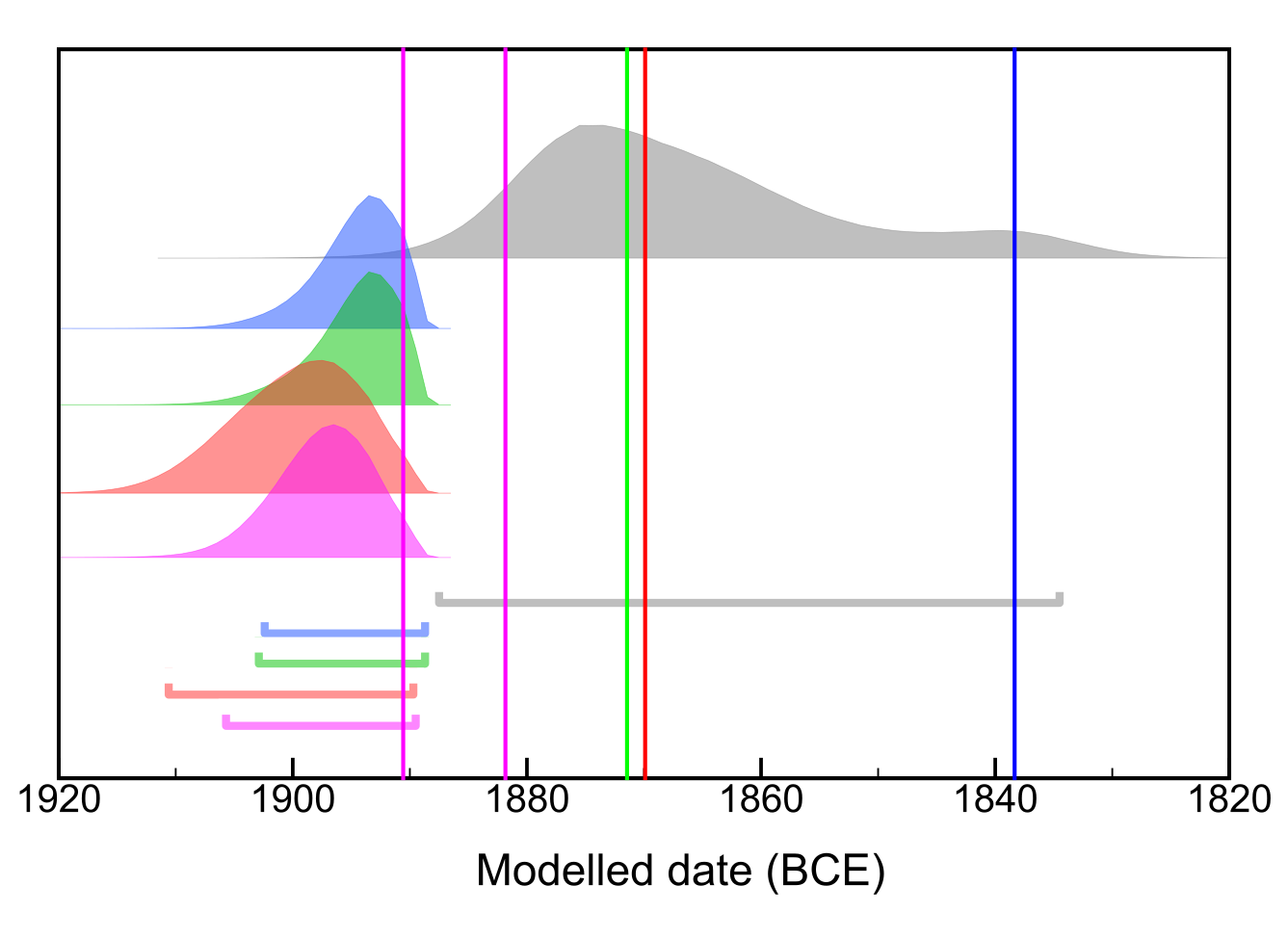

Supplement: S2 Fig — The resulting probability density functions of 4 different MK models which incorporate different reign length interpretations: Hornung et al. [21] (blue), Kitchen [4] (green), Shaw [59] (red), Gautschy [25] (two options, magenta) with horizontal bars indicating the 95.4% range. Vertical lines indicate the traditional absolute dates for the accession of Senusret III, using the same colours for the respective reign length schemes. (PNG) [file pone.0314612.s002.png]

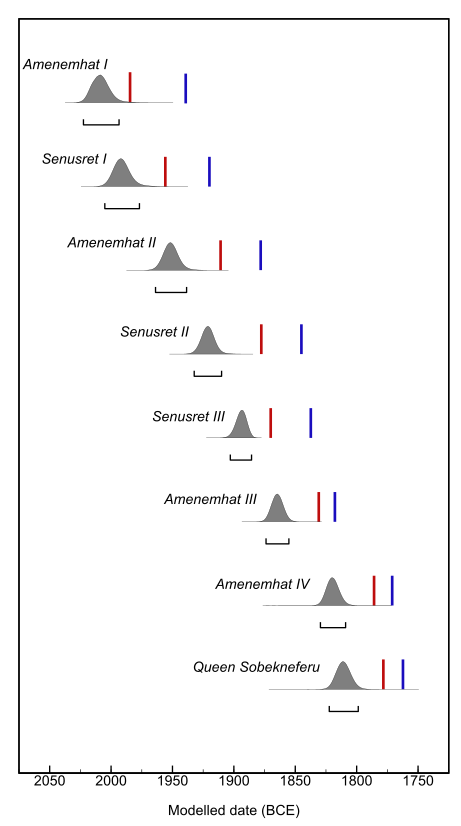

Supplement: S3 Fig — 95.4% probability ranges are shown. Traditional absolute dates for the rulers’ accession based on High [59] and Low [21] chronologies is shown for comparison as vertical bars (red and blue respectively). The probability density functions when the model is calibrated against IntCal20. (PNG) [file pone.0314612.s003.png]
