# Supplementary material for: Resolution of the High versus Low debate for Old and Middle Kingdom Egypt
Source: PLoS One. 2025 May 28;20(5):e0314612. doi: 10.1371/journal.pone.0314612 (PMC12119019; doi:10.1371/journal.pone.0314612)
Supplement: S1 Table — [43], and two published results on the Illahun Sothic Papyrus (OxA-23170 and OxA-23171) [32] listed stratigraphically. Our models incorporated the prior assumption that these documents could be regarded as forming a relative sequence in each reign, based on the inscribed regnal years. (PDF) [file pone.0314612.s004.pdf]

S1 Table. The Illahun papyri dated by Bronk Ramsey et al. (43), and two published results on the Illahun Sothic Papyrus (OxA-23170 and OxA-23171, ref. 32) listed stratigraphically. Our models incorporated the prior assumption that these documents could be regarded as forming a relative sequence in each reign, based on the inscribed regnal years.

Laboratory Code	^{14}C Date		Assignment in Model
	^{14}C yr BP	± 1σ	
OxA-15313	3503	30	Senusret III Year 5
VERA-3726	3543	29	
OxA-15317	3532	31	Senusret III Year 5
VERA-3730	3529	29	
OxA-15318	3518	31	Senusret III Year 5
VERA-3731	3513	29	
OxA-23170	3520	28	Senusret III Year 7
OxA-23171	3508	27	
OxA-15315	3513	31	Senusret III Year14
VERA-3728	3565	29	
OxA-15311	3532	31	Senusret III Year 14
VERA-3732	3563	29	Senusret III Year14
OxA-15316	3542	30	Amenemhat III Year 4
VERA-3729	3563	29	
OxA-15310	3560	33	Amenemhat III Year 4
VERA-3733	3568	28	Amenemhat III Year 4
VERA-3734	3512	29	Amenemhat III Year 37
OxA-15309	3626	33	Amenemhat III Year 37
OxA-15312	3485	31	Amenemhat III Year 38
VERA-3735	3550	29	Amenemhat III Year 40
