# Supplementary material for: Resolution of the High versus Low debate for Old and Middle Kingdom Egypt
Source: PLoS One. 2025 May 28;20(5):e0314612. doi: 10.1371/journal.pone.0314612 (PMC12119019; doi:10.1371/journal.pone.0314612)
Supplement: S2 Table — Models calibrated with IntCal20. (PDF) [file pone.0314612.s005.pdf]

**S2 Table. The modelled 95% ranges for OK rulers.** Models calibrated with IntCal20.

| | OK-P1 (ref. 21) | | | OK-P2 (ref. 4) | | | OK-P3 (ref. 55) | | | OK-P4 (ref. 26, HIGH) | | | OK-P5 (ref. 26, LOW) | | |
|---|---|---|---|---|---|---|---|---|---|---|---|---|---|---|---|
| | Accession Date (BCE, 95%) | | | Accession Date (BCE, 95%) | | | Accession Date (BCE, 95%) | | | Accession Date (BCE, 95%) | | | Accession Date (BCE, 95%) | | |
| | From | To | Median | From | To | Median | From | To | Median | From | To | Median | From | To | Median |
| Khasekhemwy | 2725 | 2635 | 2678 | 2766 | 2644 | 2693 | 2746 | 2639 | 2684 | – | – | – | – | – | – |
| Djoser (Start of OK) | 2698 | 2629 | 2662 | 2750 | 2635 | 2675 | 2704 | 2632 | 2669 | – | – | – | – | – | – |
| Sekhemkhet | 2673 | 2605 | 2637 | 2732 | 2620 | 2658 | 2687 | 2617 | 2652 | – | – | – | – | – | – |
| Sneferu | 2656 | 2587 | 2621 | 2673 | 2582 | 2618 | 2665 | 2590 | 2628 | – | – | – | – | – | – |
| Khufu | 2625 | 2553 | 2589 | 2650 | 2559 | 2597 | 2646 | 2570 | 2606 | 2667 | 2585 | 2632 | 2668 | 2585 | 2632 |
| Djedefra | 2602 | 2528 | 2565 | 2629 | 2539 | 2577 | 2625 | 2550 | 2586 | 2638 | 2561 | 2603 | 2639 | 2564 | 2603 |
| Khafra | 2593 | 2519 | 2556 | 2621 | 2532 | 2570 | 2617 | 2543 | 2579 | 2629 | 2552 | 2593 | 2630 | 2555 | 2594 |
| Menkaura | 2571 | 2498 | 2533 | 2597 | 2510 | 2549 | 2594 | 2521 | 2556 | 2603 | 2529 | 2568 | 2604 | 2530 | 2568 |
| Shepseskaf | 2564 | 2492 | 2527 | 2580 | 2495 | 2534 | 2568 | 2496 | 2530 | 2584 | 2511 | 2549 | 2585 | 2513 | 2550 |
| Userkaf | 2558 | 2486 | 2520 | 2575 | 2490 | 2529 | 2560 | 2488 | 2522 | 2575 | 2503 | 2540 | 2574 | 2503 | 2539 |
| Sahura | 2551 | 2480 | 2514 | 2567 | 2484 | 2522 | 2553 | 2483 | 2516 | 2565 | 2494 | 2530 | 2563 | 2494 | 2529 |
| Neferirkara | 2540 | 2470 | 2503 | 2554 | 2473 | 2511 | 2542 | 2474 | 2506 | 2546 | 2477 | 2512 | 2545 | 2477 | 2511 |
| Shepseskara | 2530 | 2461 | 2494 | 2544 | 2465 | 2502 | 2524 | 2458 | 2489 | 2532 | 2465 | 2499 | 2531 | 2465 | 2498 |
| Djedekara | 2500 | 2437 | 2468 | 2498 | 2434 | 2467 | 2491 | 2435 | 2464 | 2498 | 2440 | 2471 | 2495 | 2438 | 2467 |
| Unas | 2459 | 2399 | 2429 | 2461 | 2401 | 2433 | 2455 | 2403 | 2430 | 2462 | 2407 | 2437 | 2460 | 2405 | 2433 |
| Teti | 2446 | 2386 | 2417 | 2436 | 2376 | 2408 | 2429 | 2379 | 2405 | 2446 | 2389 | 2420 | 2443 | 2387 | 2416 |
| Userkara | 2424 | 2365 | 2396 | 2426 | 2367 | 2398 | 2410 | 2361 | 2387 | 2429 | 2372 | 2404 | 2426 | 2370 | 2399 |
| Pepy I | 2420 | 2361 | 2392 | 2419 | 2360 | 2392 | 2406 | 2357 | 2383 | 2425 | 2367 | 2399 | 2422 | 2366 | 2396 |
| Merenra | 2374 | 2315 | 2351 | 2377 | 2317 | 2353 | 2376 | 2327 | 2355 | 2378 | 2319 | 2354 | 2380 | 2322 | 2355 |
| Pepy II | 2366 | 2305 | 2342 | 2368 | 2308 | 2345 | 2369 | 2317 | 2347 | 2370 | 2309 | 2346 | 2370 | 2311 | 2345 |
| Late 6th Dynasty | 2311 | 2240 | 2280 | 2280 | 2214 | 2253 | 2282 | 2223 | 2256 | – | – | – | – | – | – |
| Start 7th Dynasty | 2307 | 2235 | 2275 | 2270 | 2198 | 2239 | 2278 | 2218 | 2251 | – | – | – | – | – | – |
| Start FIP | 2283 | 2204 | 2246 | 2236 | 2158 | 2201 | 2262 | 2197 | 2232 | 2322 | 2256 | 2292 | 2321 | 2257 | 2291 |
