# Supplementary material for: Resolution of the High versus Low debate for Old and Middle Kingdom Egypt
Source: PLoS One. 2025 May 28;20(5):e0314612. doi: 10.1371/journal.pone.0314612 (PMC12119019; doi:10.1371/journal.pone.0314612)
Supplement: S3 Table — Models calibrated with IntCal20. (PDF) [file pone.0314612.s006.pdf]

S3 Table. The modelled 95% ranges for MK rulers. Models calibrated with IntCal20.

	MK P1 (ref. 21)			MK P2 (ref. 4)			MK P3 (ref. 55)			MK P4 (ref. 25)		
	Accession Date (BCE, 95%)			Accession Date (BCE, 95%)			Accession Date (BCE, 95%)			Accession Date (BCE, 95%)		
	**From**	**To**	**Median**	**From**	**To**	**Median**	**From**	**To**	**Median**	**From**	**To**	**Median**
Mentuhotep II (Start of MK)	2065	2037	2051	2064	2038	2051	2070	2040	2055	–	–	–
Mentuhotep III	2013	1989	2001	2013	1989	2001	2019	1992	2006	–	–	–
Mentuhotep IV	2005	1980	1992	2003	1979	1991	2011	1982	1997	–	–	–
Amenemhat I	1998	1973	1986	1997	1973	1985	2007	1976	1992	2024	1995	2010
Senusret I	1981	1957	1969	1979	1956	1967	1985	1950	1969	2007	1978	1993
Amenemhat II	1940	1918	1929	1938	1917	1928	1948	1915	1931	1965	1940	1953
Senusret II	1907	1889	1898	1905	1888	1896	1914	1889	1900	1934	1912	1923
Senusret III	1898	1885	1891	1898	1885	1891	1908	1886	1895	1904	1887	1895
Amenemhat III	1880	1864	1872	1880	1864	1872	1872	1846	1858	1875	1857	1866
Amenemhat IV	1835	1815	1826	1835	1816	1826	1828	1800	1815	1831	1811	1821
Queen Sobekneferu	1828	1805	1817	1828	1806	1817	1822	1790	1806	1824	1800	1812
Wegaf (End of MK)	1825	1799	1813	1825	1800	1813	1818	1784	1802	–	–	–
Sobekhotep II	1823	1723	1797	1822	1726	1797	1816	1706	1786	–	–	–
