# Supplementary material for: Resolution of the High versus Low debate for Old and Middle Kingdom Egypt
Source: PLoS One. 2025 May 28;20(5):e0314612. doi: 10.1371/journal.pone.0314612 (PMC12119019; doi:10.1371/journal.pone.0314612)
Supplement: S1 File — (DOCX) [file pone.0314612.s007.docx]

Supplementary Information (File S1) for:

**Resolution of the High versus Low Debate for Old and Middle Kingdom Egypt**

Pınar Erdil^*^, Lyndelle Webster, Margot Kuitems, Christian Knoblauch, Laurel Bestock, Felix Höflmayer, Hans Beeckman, Dorian Q. Fuller, Sturt W. Manning, Michael W. Dee

*Corresponding author. Email: [p.erdil@rug.nl](mailto:p.erdil@rug.nl)

Supplementary Text:

**Materials**

**New OK Materials
 New MK Materials**

**Methods**

**Experimental
 Bayesian Modeling
 Revised OK Models
 Revised MK Models
 Use of the seasonal offset**

**OxCal Codes**

**References**

# Materials

## New OK Samples

The sampling objective for the OK partly involved filling the gaps in the models of Bronk Ramsey et al. [43], in particular the latter half of the historical period. A list of the materials is given in Table 1. Care was taken to identify samples that were both short lived and unlikely to have been reused in antiquity. Only objects supported by the best curatorial and archaeological provenance records were selected. Nevertheless, the historical attributions of these archaeological materials are sometimes open to interpretation, resulting in uncertainty. These uncertainties do not compromise the overall conclusions of our study. For most of these new samples, we have opted for broader allocations rather than assigning them to a specific reign, in same manner as the previously established ^14^C models on OK [64]. Some of them are positioned within a span covering multiple dynasties over several centuries (see Table 1, Historical Assignment). We based these allocations on the attributions provided by the initial excavators, museum curators, as well as our own Egyptological expertise.

## New MK Samples

The main focus for the sampling program was on resolving the High versus Low dichotomy that still hampers analysis of the MK (and we refer particularly to the earlier MK, the Dynasty XII —and do not discuss the later MK or Dynasty XIII). Our strategy centered on the accession date of Senusret III of Dynasty XII. Targeting this point in time was crucial for three main reasons. Firstly, the Sothic Papyrus from Illahun, upon which the MK chronology depends, lies within Senusret III's reign. Therefore, achieving a high-precision ^14^C date for his accession year would help secure both the age of the papyrus, and the position of the MK. Two ^14^C dates on the Sothic Papyrus were recently published [32], but in isolation they are too broad to be of real value. A further 46 measurements from the cedar wood funerary boat at the pyramid complex of Senusret III were also published by Manning et al. [46]. Thirdly, tree-ring sequences and short-lived samples also became available from the Nubian fortress of Uronarti, constructed within Senusret III’s reign. The combination of ^14^C dating and tree-ring sequences can sometimes offer extremely precise results [65, 66]. Uronarti is historically dated to Year 16 of Senusret III, courtesy of a stela found at the site [67, 68]. Thus, collectively, the means were available for determining whether Senusret III’s reign lay in the first (High Chronology) or second (Low Chronology) half of the 19^th^ century BCE.

The MK fortress of Uronarti is located in the region of Lower Nubia within the borders of modern-day Sudan (21.525833 N, 30.990278 E) [67-69]. It is a triangular-shaped fortress with barracks, granaries, workshops, and administrative buildings built on an island on the Nile. [67, 68, 70, 71]. Since 2012, excavations have been carried out by the Uronarti Regional Archaeological Project (URAP) team led by Brown and Swansea Universities [49, 72-74]. In collaboration with the URAP team, two wooden support beams and short-lived plants were sampled from the fortress (listed in Tables 2-3).

One tree beam (code: FK-001-01) was obtained from the primary entrance of the fortress: South Gate; whereas the second one (code: FJ-001-04) was from the outer wall, the East Wall North. Both were strengthening elements embedded in the original brickwork as was common for all MK Nubian fortresses [75]. The preservation state of these wood samples was particularly striking (see S1 Fig). The tree species was identified as *Vachellia tortilis* (also known as *Acacia tortilis*) by Prof. Dr. Pieter Baas at Naturalis Biodiversity Center, Leiden. The provenance of these tree beams is difficult to prove. However, the species grows over a large geographical area in northern Africa and is considered to be a keystone species in this region [76]. Since the species is commonly found where the fortress is located, we have no reason to believe that the timber was sourced from outside of modern-day Egypt or Sudan. Past research on this species indicates that tree-ring formation may or may not be annual [77-81]. Physiological analysis of the beams did not indicate any missing rings; however, the dendrochronological analysis was inconclusive. The results of the tree-ring measurements are given in Table 3.

# Methods

## Experimental

The OK samples were pretreated and dated at the Oxford Radiocarbon Accelerator Unit (ORAU). The associated procedures can be found in Brock et al. [48]. In summary, the bone samples were subject to routine acid-base-acid applications followed by gelatinization and ultrafiltration. The short-lived plants, including items such as linen and papyrus, were pretreated using an acid-base-acid sequence, but also followed by an oxidation with NaClO_2_. For sturdier samples, the oxidant was applied at 5% w/vol concentration and for fragile samples, 2.5% w/vol. The subsequent gelatin (bone) and holocellulose (plant) extracts were freeze-dried, combusted, graphitized and measured by accelerator mass spectrometry (AMS).

The physical and chemical pretreatment steps for the two transverse sections of wood and the short-lived plant samples from Uronarti were completed at the Centre for Isotope Research (CIO), University of Groningen. Only 10 and 7 growth rings could be identified for FK-001-01 and FJ-001-04, respectively. Subsamples 1-2 cm in size were obtained for each ring, by cutting with a scalpel around the ring borders. All the wood samples from Uronarti were subjected to the intensive α-cellulose protocol, whereas the short-lived samples were subjected to the holocellulose extraction protocol employed by the CIO [50]. Subsequently, all samples were freeze-dried, combusted to CO_2_, graphitized and measured by AMS at the CIO. Each of these steps is also described in detail in Dee et al. [50].

## Bayesian modelling

We prepared new Bayesian models for OK and MK, based on those published by Bronk Ramsey et al. [43] but incorporating several new developments (OxCal command terms are shown below in Courier font, e.g.: Sequence or Phase):

### Revised OK models

For the OK, we updated the configurations of Boundaries and Phases to incorporate reigns that could not previously be included in the 2010 models. For example, the accession dates of Neferirkara, Shepseskara, and Pepy II were not included in the original model configuration due to lack of sufficient samples to distinguish their reigns. However, 5 new ^14^C dates from the reign of Pepy II, recently published by Quiles et al. [82], along with a new date from the reign of his predecessor Merenra (this paper), have allowed us to identify the accession date of Pepy II. Similarly, a new ^14^C date from the reign of Neferirkara enabled us to include the reigns of both Neferirkara and his successor Shepseskara.

We followed the modelling configurations and specifications established by Bronk Ramsey et al. [43] and Dee [64], which allow for a high degree of flexibility in the final position of the modelled ^14^C dates. Their approach ensures that the models can accommodate the uncertainty in sample allocation. The precision of all the outputs is then a product of both the tightly assigned samples and, to a much lesser extent, the more broadly defined ones.

### Revised MK models

For the MK, the key differences were the incorporation of a Sequence model for the tree-ring and short-lived plants from the fortress of Uronarti; the funerary boat measurements; and the use we made of the ordering of the Illahun papyrus dates which all fell under the reign of Senusret III. Most of the following updates to the MK models relate to the reign of Senusret III.

First, using the results on the two transverse sections of wood, simple models were run using the Sequence function in OxCal. Separate models were run for the two beams (OxCal code for FK-001-01: URO1, OxCal code for FJ-001-04: URO2). Since we did not know whether the ring formations were annual or not, we opted to place the tree-rings in a simple Sequence model without any Gap or Interval functions in between. The modelled ^14^C dates we obtained for the bark edges (GrM-22702 and GrM-24029) corresponded with the construction of the fortress (see assumptions mentioned in ‘Materials, New MK Samples’) and this information was included in the MK models within the reign of Senusret III. To be precise, the estimated felling dates were placed within the Phase of the model representing his reign. We also opted not to regard the felling date of the wood samples as exactly corresponding to Year 16 of Senusret III (the regnal year that appears on the stela found at Uronarti). This could be a dedication date, following completion of the fortress, and the stela might have been erected some years after the trees were cut.

The short-lived plant samples from Uronarti were obtained from a stratified sequence, which is listed in Table 2 from the earliest to the latest stage of the occupation. The ^14^C dates from the same stratigraphy are grouped in a Phase with defined Boundaries between each Phase. These ^14^C dates were incorporated into a simple Sequence model (OxCal code: URO3), allowing us to achieve higher precision for the foundation Boundary. The modelled date of this foundation Boundary of was then included in the MK model under the Phase for Senusret III, since the start of construction would have fallen within his reign.

Additionally, 49 high-precision ^14^C measurements on the tree-rings from the funerary boat found at the pyramid complex of Senusret III were published by Manning et al. [46]. The analysis of the wood suggested that the origin of the timbers was Lebanon [46]. The reader is referred to Manning et al. [46] for further information on this analysis. The felling date of this sequence was placed within the Phase representing Senusret III’s reign as a *terminus post quem* only, using the After function on OxCal.

It is advisable to note that these Sequences (URO1, URO2 and URO3 as well as the funerary boat Sequence from ref. 46) are best run separately in OxCal. The modelled bark edges from the tree-ring Sequences, the foundation Boundary from the short-lived plant Sequence and the felling date Boundary from the boat Sequence are best then saved as Priors and placed into the MK models using the Insert/Prior function. This method ensures to get functional runs of OxCal, so that the program can converge and complete each individual code without being affected by computational limitations.

Lastly, we have enhanced the use of the dates on the Illahun papyri cache which were previously included in the models of Bronk Ramsey et al. [43]. No ordering of these documents was used in their models. These papyri are examples of precise record keeping by the Egyptians and include the information on the regnal year of the reigns in which they were written. A total of 19 ^14^C dates on such papyri are available (including the two new measurements from Marcus et al. [32] on Illahun papyrus corresponding to Year 7 of Senusret III since the 2010 publication) relates to different years from the reigns of Senusret III and Amenemhet III, and hence the documents can easily be arranged in relative chronological order (see S1 Table).

We might also have assumed that papyri nominally from the same year were harvested and used in that year. In such a scenario, a D_Sequence could have been employed, with prescribed Intervals (number of regnal years) between each group of documents. However, it is also possible that any given papyrus was a year or two old when it was used (or even re-used)^^[[1]](#footnote-1)^^. So, instead, the dates were included within bounded Phases representing their regnal year (Year 5, 7, 14 for Senusret III and Year 4, 37, 38 and 40 for Amenemhet III). These bounded Phases were nested in turn within a Sequence inside the Phase for each king's reign

### Use of the Seasonal Offset

The Egyptian seasonal offset (12 ± 5 ^14^C yr BP based on IntCal20) was deemed inappropriate for the tree-ring Sequences for Uronarti and the funerary boat. The offset originally reported by Dee et al. [45] is attributed to a growing season effect in Egypt, whereby plant growth was dependent on the annual flooding of the Nile. As the flood occurred between June and October, plants in the Nile Valley and Delta in antiquity tended to grow between winter and spring. This is the exact opposite of the tree rings used to construct the Northern Hemisphere calibration curve, which grows between spring and summer. These two periods (spring to summer and winter to spring) in the Northern Hemisphere are known to have slightly different atmospheric ^14^C levels [45].

The timber beams found on Uronarti are identified as *Acacia tortilis*. In arid climates, these species are known to have a deep root system, which allows the trees to access groundwater all year round [83]. They are observed to grow all year round (even in hyper-arid conditions) and growth is unrelated to rainfall or flashflood events [84]. Therefore, the growing season for Acacia species is typically long and trees are able to use water for at least 10-11 months. The growth rings are usually produced when the tree has a short leafless period in winter. Since the trees from Uronarti could grow all year, they would not have experienced a growing season effect. Therefore, there is no reason to account for a seasonal offset. For the funerary boat, the timber does not originate from Egypt, but instead likely from the mountains of Lebanon. In the mountains of northern Lebanon, cedar growth is usually later spring through summer and thus approximately parallel with the trees used for IntCal, but specimens from lower elevations or other areas may vary somewhat. Comparison of the wiggle-match from the funerary boat found at the pyramid complex of Senusret III against IntCal20 with a neutral prior Delta_R of 0 ± 10 ^14^C years suggests an offset of 13 ± 5 ^14^C years. Comparisons of other Lebanon cedar series against previous versions of IntCal indicate variable findings with some offset or no apparent offset at different periods [46].

However, the seasonal offset was applied to the short-lived plant sequence from Uronarti as well as the OK and MK models. The OK and MK models are mostly made up of ^14^C dates on short-lived plants and these would be prone to the growing season of Egypt where an offset could indeed affect the dates. The Delta_R command in OxCal was used in these models to add 12 ± 5 ^14^C yr BP as a prior. As a test, we also ran the MK models without any offset applied. That is, we ran all the tree-ring Sequences, short-lived plant Sequences and the MK models without a Delta_R command at all. The results for the probability of king Senusret III’s reign are given in S2 Fig. In sum, the difference between models which applied the seasonal effect and the ones that did not is negligible. In the main text, the models ran with IntCal20 have the 12 ± 5 ^14^C yr BP as a prior applied.

*Reign-length Configurations:* As in Shortland and Bronk Ramsey [85], we used the Interval command to denote the reign-length estimates proposed by Hornung et al. [21], Kitchen [4], and Shaw [55]. We also include two recent publications on reign-length estimates by Gautschy et al. (ref.26; OK reign lengths with both High and Low chronology options) and Gautschy (ref.25; MK reign lengths). These reign-length differences necessitated the construction of five distinct models for OK and four models for MK: OK-P1 & MK-P1 [21], OK-P1 & MK-2 [4], OK-P1 & MK-P3 [55], OK-P4 (High chronology, ref.26), OK-P5 (Low chronology, ref.26), MK-P4 [25]. The modelled date ranges for OK and MK rulers are given in S2 and S3 Tables. We chose to depict the results of the MK model with the most recent publication on reign lengths [25] and plotted the probability graphs of Dynasty XII rulers in S3 Fig.

# OxCal Codes

All codes are available in GitHub repository: <https://github.com/pinarerdil/OKMK.git>

# References

1. Dee, MW. A Radiocarbon-based Chronology for the Old Kingdom. In: Shortland AJ, Bronk Ramsey C, editors. Radiocarbon and the chronologies of ancient Egypt. Oxbow Books; 2013. pp. 298-311
2. Kuitems M, Panin A, Scifo A, Arzhantseva I, Kononov Y, Doeve P, et al. Radiocarbon-based approach capable of subannual precision resolves the origins of the site of Por-Bajin. Proc Natl Acad Sci USA. 2020;117: 14038-14041.
3. Kuitems M, Wallace BL, Lindsay C, Scifo A, Doeve P, Jenkins K, et al. Evidence for European presence in the Americas in AD 1021. Nature. 2022;601: 388-391.
4. Dunham D, Janssen JMA. Second Cataract Forts Volume II: Uronarti, Shalfak, Mirgissa excavated by George Andrew Reisner, and Noel F. Wheeler. Boston Museum of Fine Arts; 1967.
5. van Siclen CC III. The Chapel of Sesostris III at Uronarti. Van Siclen; 1982.
6. Clarke S. Ancient Egyptian frontier fortresses. JEA. 1916;3: 155-79.
7. Vogel C, Delf B. The fortifications of ancient Egypt, 3000-1780 BC (Fortress). Osprey; 2010.
8. Bestock L. Forgotten fortress: returning to Uronarti. NEA. 2017;80: 154-165.
9. Bestock L, Knoblauch C. Revisiting Middle Kingdom interactions in Nubia: the Uronarti Regional Archaeological Project. JAEI. 2014;6: 32-35.
10. Knoblauch C, Bestock L. The Uronarti Regional Archaeological Project: final report of the 2012 survey. MDAIK. 2013;69: 103-142.
11. Knoblauch C, Bestock L. Evolving communities: the Egyptian fortress on Uronarti in the late Middle Kingdom. Sudan & Nubia. 2017;21: 50-58.
12. Näser C, Becker P, Kossatz K, Karrar OKE, Grajetzki W. Shalfak Archaeological Mission (SAM): the 2017 field season. JEA. 2017;103: 153-171.
13. Noumi Z, Chaieb M. Dynamics of Acacia tortilis (Forssk.) Hayne subsp. raddiana (Savi) Brenan in arid zones of Tunisia. Acta Bot Gallica. 2012;159: 121-126.
14. Wyant JG, Reid RS. Determining the age of Acacia tortilis with ring counts for South Turkana, Kenya: a preliminary assessment. Afr J Ecol. 1992;30: 176-180.
15. Gourlay ID. Growth ring characteristics of some African Acacia species. J Trop Ecol. 1995;11: 121-140.
16. Martin D, Moss J. Age determination of Acacia tortilis (Forsk.) Hayne from northern Kenya. Afr J Ecol. 1997;35: 266-277.
17. Andersen GL, Krzywinski K. Longevity and growth of Acacia tortilis: insights from 14C content and anatomy of wood. BMC Ecol. 2007;7: 1-14.
18. Gebrekirstos A, Mitlöhner R, Teketay D, Worbes M. Climate–growth relationships of the dominant tree species from semi-arid savanna woodland in Ethiopia. Trees. 2008;22: 631-641.
19. Quiles A, Sowada K, Kanawati N. Dating the end of the Egyptian Old Kingdom: new contextualized dates from the reign of king Pepy II. Radiocarbon. 2023;65: 1080-1097.
20. Orwa C, Mutua A, Kindt R, Jamnadass R, Simons A. Agroforestree Database: a tree reference and selection guide version 4.0 World Agroforestry Centre, Kenya; 2009 [cited 2023 June 13]. Available from: https://www.worldagroforestry.org/output/agroforestree-database.
21. Winters G, Otieno D, Cohen S, Bogner C, Ragowloski G, Paudel I, Klein T. Tree growth and water-use in hyper-arid Acacia occurs during the hottest and driest season. Oecologia. 2018;188: 695-705.
22. Shortland AJ, Bronk Ramsey C, editors. Radiocarbon and the chronologies of ancient Egypt. Oxbow Books; 2013.

1. Palimpsests also occur in which papyri were reused generations or even centuries after their original manufacture. However, we were not alerted to any signs of reuse or erasure in this case. [↑](#footnote-ref-1)
